# Exploring the feasibility of Zr-based metal–organic frameworks for the recovery of Sb (V) and Sb (III) from mining waste

**DOI:** 10.1038/s41598-024-65360-2

**Published:** 2024-07-08

**Authors:** Daolu Bu, Hu Yang, Haibo Zhang, Liang Wang, Jiao Wang, Jie Liao

**Affiliations:** 1 Research Center of Applied Geology of China Geological Survey, Chengdu, China; 2 Zhejiang ECO Environmental Protection Technology Co., LTD, Huzhou, China

**Keywords:** Metal–organic frameworks, Wastewater management, Antimony separation, Mining wastewater, Zr-based MOFs, Environmental sciences, Chemistry, Chemical engineering, Materials for energy and catalysis

## Abstract

The present study investigates the efficacy of newly developed Zr-based metal–organic frameworks, specifically MIP-206, and its amine-modified counterpart, MIP-206-NH_2_, for the re-covery of antimony (Sb) from both synthetic and actual mining wastewater. Batch method studies were employed to examine the effect of waste media pH, Sb concentration, process kinetics, and the performance of the regeneration solution. MIP-206-NH_2_ exhibited impressive separation capabilities, achieving 102.18 mg/g and 63.23 mg/g for Sb (V) and Sb (III), respectively. In contrast, the pristine MIP-206 reached maximum values of 26.26 mg/g for Sb (V) and 16.95 mg/g for Sb (III). The separation process was well-described by the Langmuir equation, and the kinetics followed the pseudo-second-order model. Although the amine modification resulted in a decrease in the surface area of MIP-206 from 1345.21 to 1169.86 m^2^/g, SEM and XRD analyses confirmed that the structural integrity of MIP-206-NH_2_ remained unchanged. In terms of reusability, MIP-206-NH_2_ maintained up to 90% of its separation performance over 9 cycles, while MIP-206 demonstrated effectiveness for 7 cycles. The regeneration solution exhibited a capacity of approximately 0.63 mol/L for Sb (V) and 0.71 mol/L for Sb (III). Furthermore, MIP-206 and MIP-206-NH_2_ demonstrated successful application in selectively separating Sb from real mining wastewater.

## Introduction

**Figure 1:**
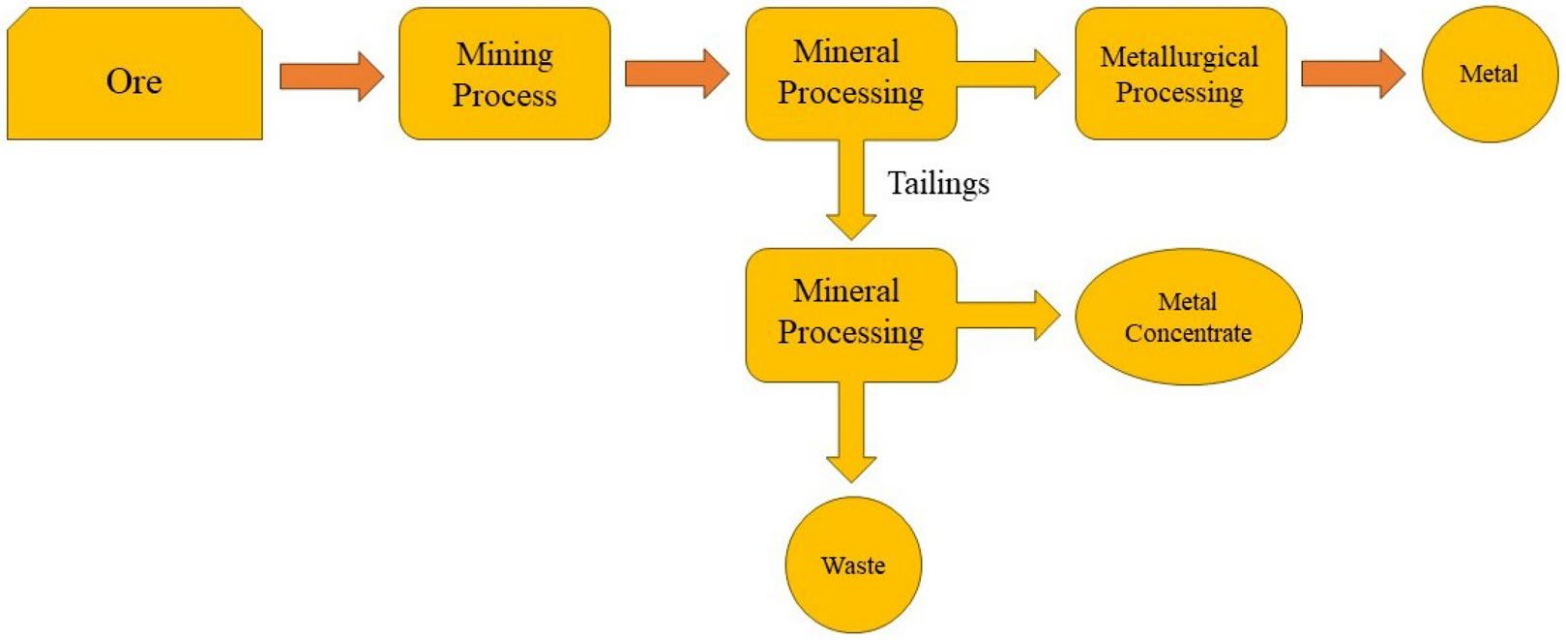
Schematic illustration of metal recovery process from waste sources.

The concept of a circular economy has been conceptualized with the aim of finding tangible answers to attain a sustainable economy^1^. A pivotal aspect involves the adept handling of metals to bolster resource recycling^2,3^. In this context, the valorization of waste materials emerges as a pivotal element for the world-wide circular economy^4–6^. This strategy offers dual benefits: the purification of waste, leading to a subsequent reduction in the overall toxicity of disposables, and the retrieval of metals that would otherwise contribute to the composition of waste materials^7–10^. Wastewater of mine tailings generated during the mineral extraction process in different sectors of the metal industry might be harnessed as a source in a procedure engineered to procure one or more essential raw materials. A simplified diagram outlining this concept is depicted in Fig. 1.

Antimony (Sb) ranks as the ninth most extracted element globally, emphasizing the necessity to explore both its inherent value and the potential environmental risks associated with its presence in tailings wastewater^11^. A case in point is the renowned antimony mining region of Xikuang Mountain in Hunan, China. Here, the concentrations of dissolved antimony in tailings were measured between 4.58 and 29.4 mg/L, surpassing the prescribed maximum discharge limitation of 0.3 mg/L for mining wastewater^12^. This disparity underscores the urgent necessity to formulate ap-proaches for isolating antimony from tailing wastewater, aiming either at its recovery or efficient removal from the waste stream.

Typically, the inorganic states of antimony, namely Sb(III) and Sb(V), are identified in waste media based on the prevailing pH^13,14^. In oxidizing conditions within the pH range of 3 to 10, antimony takes the form of Sb(OH)_6_^-^, indicating Sb(V), while Sb(OH)_3_ predominantly represents the form of Sb(III)^15^. In highly acidic conditions, such as pH below 2, Sb(V) appears as SbO^2+^, and within the pH range of 2–2.7 H_3_SbO_4_ is the dominant form^16^. Given the intricacy of its presence in waste media, it is crucial to employ an effective method for isolating antimony from this medium. Membrane filtration, reverse osmosis, electrodeposition, ion exchange, and adsorption represent a subset of the techniques frequently applied to eliminate antimony from waste media^17,18^. Within these methodologies, adsorption is recognized as a secure, un-complicated, cost-effective, swift, space-efficient, and environmentally friendly technique for the separation^19,20^.

Until now, materials such as multiwalled carbon nanotubes, Fe–Mn binary oxide, and graphene have frequently been utilized in the removal of Sb(III)^21–23^. However, there has been limited exploration in the development of adsorbents designed for the concurrent separation of both Sb(V) and Sb(III) compounds. An example is an adsorbent developed by Zhao et al., PVA-Fe0, which exhibited separation performance of merely 1.65 mg/g (Sb(V)) and 6.99 mg/g (Sb(III))^24^. Similarly, Luo et al. found that ZCN exhibited separation capabilities of 57.17 mg/g (Sb(V)) and 70.83 mg/g (Sb(III))^25^. An instance of research endeavors focusing on the isolation of a singular form of antimony is found in the study conducted by Feng et al.^26^. They engineered a material, denoted as NFO, with a separation capacity of 78.1 mg/g specifically for antimony(V)^26^. Despite these advances, there is still a major need for more efficient adsorbents for the simultaneous separation of Sb(V) and Sb(III) from waste media.

Metal–organic frameworks (MOFs) have gained considerable attention owing to their various strcutures, and pore tunability^27^. They allow for numerous potential applications such as linear optical characteristics, chemical sensing, and separation^28,29^. Recently, there has been a major enhansement in the use of MOFs for the effective separation of hazardous elements such as aromatic compounds, alcohols, organic dyes, and heavy metal ions from waste media^30–33^. Among developed MOFs, MIL-based MOFs have been mostly used for water decontamination. However, Cr-based MILs contain chromium species that are toxic, which presents risks to both the environment and human health throughout their synthesis and usage. The pioneering synthesis of Zr-based MOFs (UiO-66) by Cavka et al.'s sparked widespread interest due to its superior chemical and thermal characteristics^34^. Zr-based MOFs circumvent environmental concerns, making them safer and more environmentally sound choices for various waste treatment applications. Current research efforts are aimed at improving the performance of Zr-based MOFs. Wang et al. recently reported on the synthesis of MIP-206, a Zr-based MOF, utilizing isophthalic acid (IPA) as the organic linker^35^. Unlike MILs, the production of MIP-206 involves more manageable conditions, rendering it a more feasible option for large-scale applications in separating antimony from mining waste. Within MIP-206, all IPA ligands adopt a uniform connection mode, orienting towards the 5-position on the inner surface. This configuration provides a suitable environment for the interactions of active sites with antimony species and enhancing adsorption performance. Wang et al. utilized MIP-206 as a support template for palladium nanoparticles in heterogeneous catalytic processes^35^. Despite its exceptional qualities, the entire range of its performance and potential across multiple areas remains mostly unknown, presenting an interesting opportunity for future research.

The aim of this research is to explore the feasibility of using Zr-based MOFs, specifically MIP-206 and its amine-functionalized variant MIP-206-NH2, for the effective separation and recovery of antimony in both Sb(V) and Sb(III) forms from synthetic and real mining waste media. This investigation aims to evaluate the performance, efficiency, and characterization of these MOFs in the context of antimony removal.

## Materials and methods

### Chemicals and reagents

Chemicals used in the current study include K_2_H_2_Sb_2_O_7_⋅4H_2_O, and K(SbO)C_4_H_4_O_6_⋅0.5H_2_O (Aladdin Chemistry), zirconium tetrachloride (Merck), formic acid (Sigma-Aldrich), tris(2-aminoethyl) amine (Sigma-Aldrich), sodium hydroxide, and hydrochloric (Sigma-Aldrich) isophthalic acid and 5-hydroxyl-isophthalic acid (Merck), ammonium hydroxide (Sigma-Aldrich). The wastewater samples used in this study were obtained from the Nandan Tea Mountain mining area located in Hechi City, Huangxi Province of China.

### Synthesis procedure of MIP-206 and MIP-206-NH_2_

**Figure 2:**
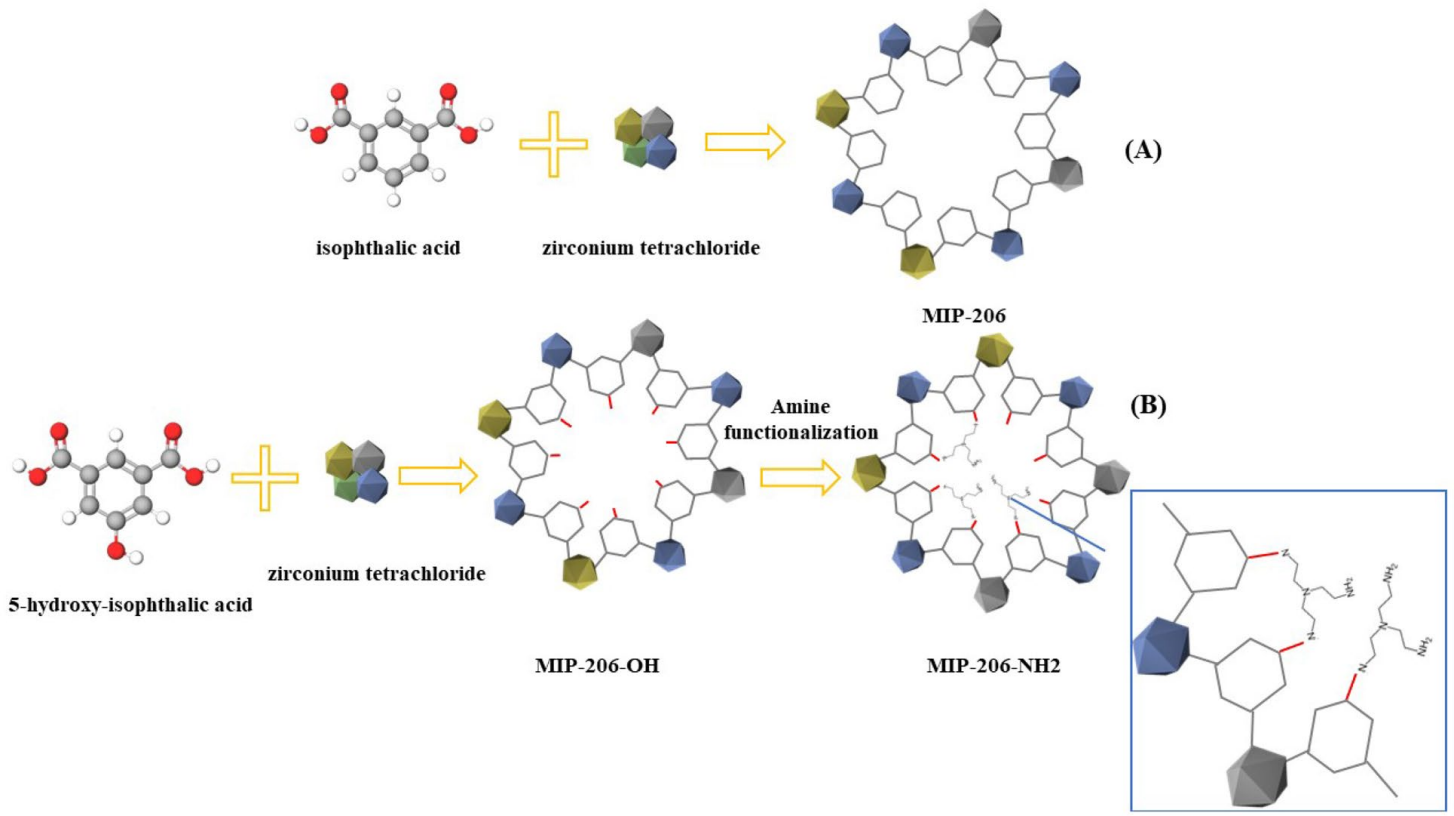
Schematic illustration of synthesis of (**A**) MIP-206, and (**B**) MIP-206-NH_2_.

The synthesis of MIP-206 followed the procedure outlined by Wang et al.^35^. In the production of amine-functionalized MIP-206, MIP-206-OH underwent amination using tris(2-aminoethyl)amine, following the identical procedure detailed in Wang et al.'s work^35^. Subsequently, the material underwent overnight vacuum heating at 120 °C. It was then mixed with a 20 mL solution of tris(2-aminoethyl) amine dissolved in CHCl3 for 45 min, with the ratio of tris(2-aminoethyl) amine to MIP-206-OH calculated stoichiometrically. The resulting material, named MIP-206-NH_2_, was even-tually isolated via centrifugation and subjected to repeated washing with CHCl3. The schematic synthesis procedure is depicted in Fig. 2.

### Instruments

A JEOL JSM-7800F model scanning electron microscope (SEM) was used for surface morphology analysis. The MIP samples were subjected to XPS studies utilizing a monochromatic Al Kα X-ray source (18.7 mA, 13.02 kV) with the SCIENCE SES 2002 instrument. A Bruker D8 Advance model X-ray diffraction (XRD) device was used for the analysis of crystal structure. The operational parameters of the device were 40 kV and 30 mA using Cu Kα radiation (λ = 1.5406 Å). The FTIR spectra of materials were collected using a PerkinElmer Spectrum Two model spectrometer. To measure the surface area of the materials a Micromeritics TriStar II Plus model gas adsorption device was used for nitrogen gas adsorption–desorption process. Barrett-Joyner-Halenda (BJH) method to calculate the pore diameter from the nitrogen gas adsorption–desorption isotherms. To measure the concentration of metal content throughout the study a 230 ATS Atomic Absorption model spectrometer (AAS) was used. The centrifuge used in the study was a benchtop 5810 R Eppendorf model device.

### Sb recovery process from synthetic wastewater media

Initially, two separate stock solutions of Sb(V) and Sb(III) with a metal content of 1000 mg/L was prepared by dissolving 2.085 g K_2_H_2_Sb_2_O_7_⋅4H_2_O and 2.743 g K(SbO)C4H4O6⋅0.5H2O in 1 L of deionized water, respectively. Synthetic waste media were then created by diluting the stock solution to achieve a Sb(V) and Sb(III) content range of 10–300 mg/L. Subsequently, 380 mg/L of either MIP-206 or MIP-206-NH_2_ was introduced to 200 mL of each Sb solutions with different concentrations. Each solution underwent stirring for 24 h to ensure equilibrium. Following the isolation of the solid material, the Sb content was measured using AAS. The performance of MIPs for Sb separation was obtained using Eq. 1.

1 $$ (Q_{e} ) = (C_{i} - C_{e} ) \times \frac{V}{m} $$

Here, Q_e_ (mg/g) is the separation performance of MIPs, C_i_ (mg/L) is the Sb amount before separation, while C_e_ (mg/L) is the residual Sb content after the recovery process. V (L) and m (g) denote the volume of synthetic waste and the weight of MIPs, respectively.

The recovery of Sb(V) and Sb(III) from MIPs was carried out using a 1 M NaOH solution. Briefly, the used MIPs were immersed in 1 L of 1 M NaOH (regeneration solution) solution for 12 h. Next, the MIPs were isolated, and an Sb-containing solution was obtained. To assess the reusability performance of the MIPs, multiple cycles of the recovery process were performed with fresh regeneration solutions, and the performance of the MIPs in separating Sb(V) and Sb(III) from waste media was assessed each time. To evaluate the effectiveness of the regeneration solution, a 1-L batch of the solution was prepared and utilized across multiple cycles with only one-time used MIPs. The content of released Sb content was measured using AAS.

### Kinetics analysis

To assess the kinetics of the process, 380 mg/L of either MIP-206 or MIP-206-NH_2_ was added to 200 mL solutions containing Sb(V) or Sb(III) at a concentration of 300 mg/L. Sampling from these mixtures was conducted at various time intervals under continuous stirring of the solutions. Following the isolation of MIPs from each sample, the remaining Sb(V) and Sb(III) content was measured using AAS.

### Analysis of pH effect on the Sb separation process

The investigation into the pH impact on the Sb separation process involved preparing a series of Sb(V) or Sb(III) solutions with varied pH values within the range of 1.5–12 using 0.1M NaOH or HCl, while maintaining an Sb content of 300 mg/L. Subsequently, 380 mg/L of either MIP-206 or MIP-206-NH_2_ was introduced to 200 mL of each solution. Each sample underwent stirring for 24 h to achieve equilibrium. Following this, the MIPs were isolated from the solutions, and the residual Sb content was measured using AAS.

### Sb recovery process from real wastewater media

A real wastewater sample containing Sb was used to assess the performance of MIPs for the separation of Sb. Typically, 380 mg/L of either MIP-206 or MIP-206-NH_2_ was introduced to 200 mL of wastewater sample. The mixture was kept under stirring for 24 h. Next, MIPs were isolated and the remaining metal content was measured using AAS.

## Results

### SEM

**Figure 3:**
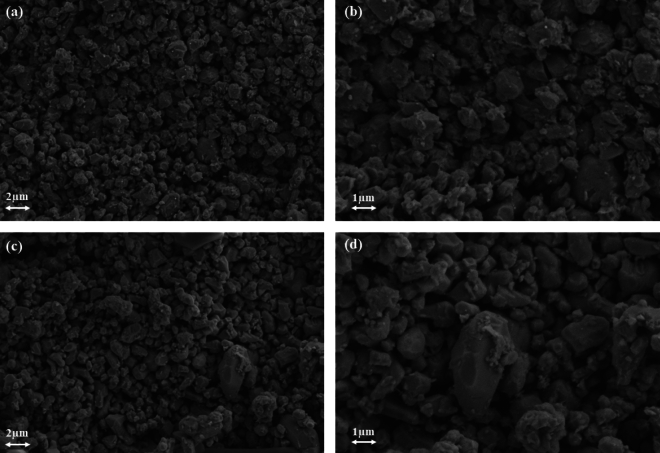
SEM images of MIP-206 (**a**) and (**b**), and MIP-206-NH_2_ (**c**) and (**d**).

Figure 3 depicts the morphology of MIP-206 and MIP-206-NH_2_. As evident from the figure, MIPs exhibit sizes approximately around 1 µm, and they appear to be isolated without any interconnected structures. Following the functionalization with amine moieties, no observable structural changes are noted. Additionally, both MIP-206 and MIP-206-NH_2_ exhibit irregular shapes with rough surfaces.

### XRD

**Figure 4:**
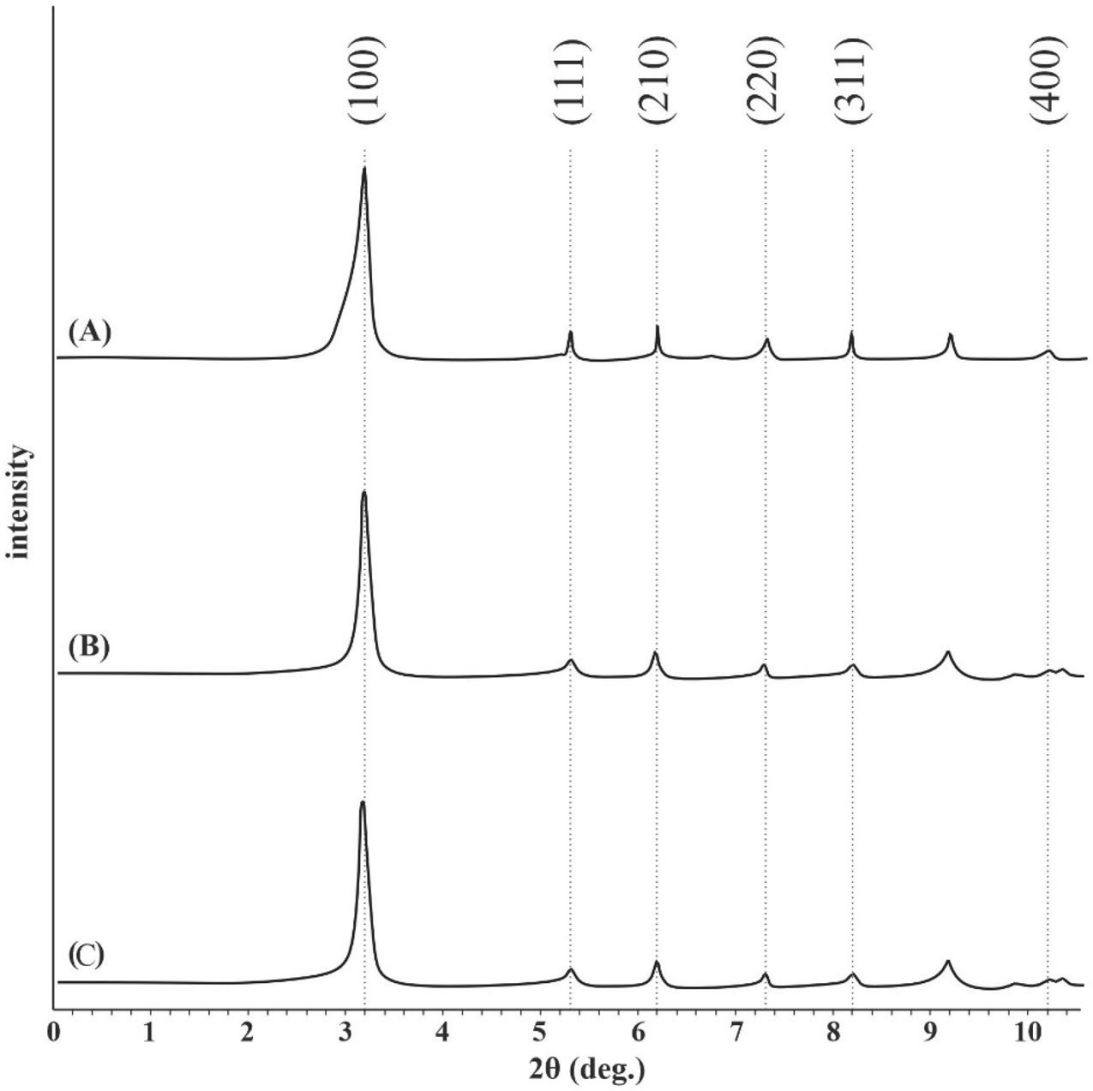
XRD spectra of (**a**) MIP-206, and (**b**) MIP-206-NH_2_, (**c**) MIP-206-NH_2_ after separation process at pH 2.

The XRD patterns for both MIP-206 and MIP-206-NH_2_ are presented in Fig. 4. The reflex positions observed at 3.1°, 5.3°, 6.2°, 7.3°, 8.2°, and 10.2° in Fig. 4a align with the characteristic reflex positions of MIP-206, confirming the successful synthesis of MIP-206 in this study^35^. Furthermore, following the functionalization with amine moieties, there are no substantial alterations in the reflex positions or their intensities (Fig. 4b), indicating the preservation of the structural integrity of the material. These findings are in accordance with those obtained from the SEM analysis. Figure 4c displays the XRD pattern of MIP-206-NH_2_ after the separation process at pH 2. Analysis of this figure indicates that the structural integrity of the material remained well preserved, even under these harsh acidic conditions, as there were no sig-nificant changes observed in the crystalline structure.

### FTIR

**Figure 5:**
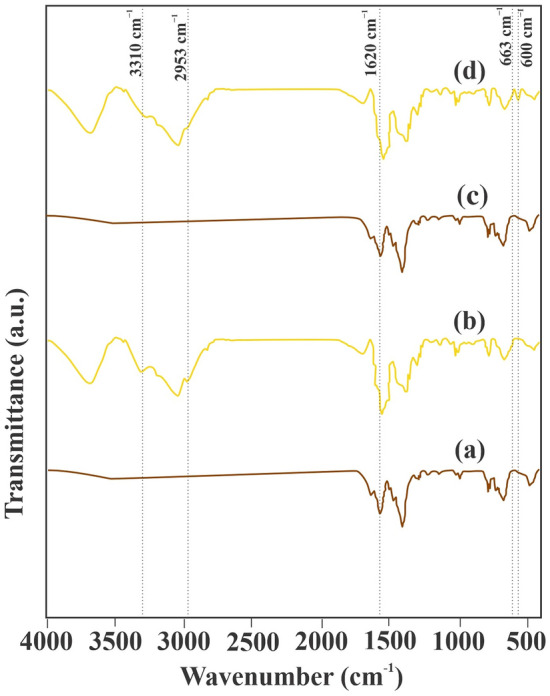
FTIR spectra of (**a**) MIP-206, (**b**) MIP-206-NH_2_, (**c**) MIP-206 after Sb ion adsorption, and (**d**) MIP-206-NH_2_ after Sb ion adsorption.

Figure 5 illustrates the FTIR spectra of MIP-206 and MIP-206-NH_2_. In Fig. 5a, peaks at 1620, 1547, and 1459 cm^−1^ correspond to aromatic C=C bonds, vibration of C–C, and vibration of C–H, respectively ^36,37^. Following amine functionalization, new peaks become evident in Fig. 5b. Peaks at 663, 2953, and 3310 cm^−1^ are attributed to N–H vibration, C–N vibration, and N–H stretching, respectively^38^. These additional peaks indicate the successful incorporation of amine moieties into the pore network of MIP-206. To explore the specific interactions between MOFs and antiomony, FTIR analysis was performed after the separation process. The peaks at 663, 2953, and 3310 cm^−1^, corresponding to N–H bending vibrations, C–N vibrations, and N–H stretching in MIP-206-NH_2_, respectively, showed a slight decrease, indicating interactions between amine groups and Sb ions. Furthermore, a peak at 600 cm^−1^, related to the bending vibration of the metal–ligand bond, emerged after metal ion removal, indicating the formation of Sb–N bonds^39^.

### Surface area analysis

**Figure 6:**
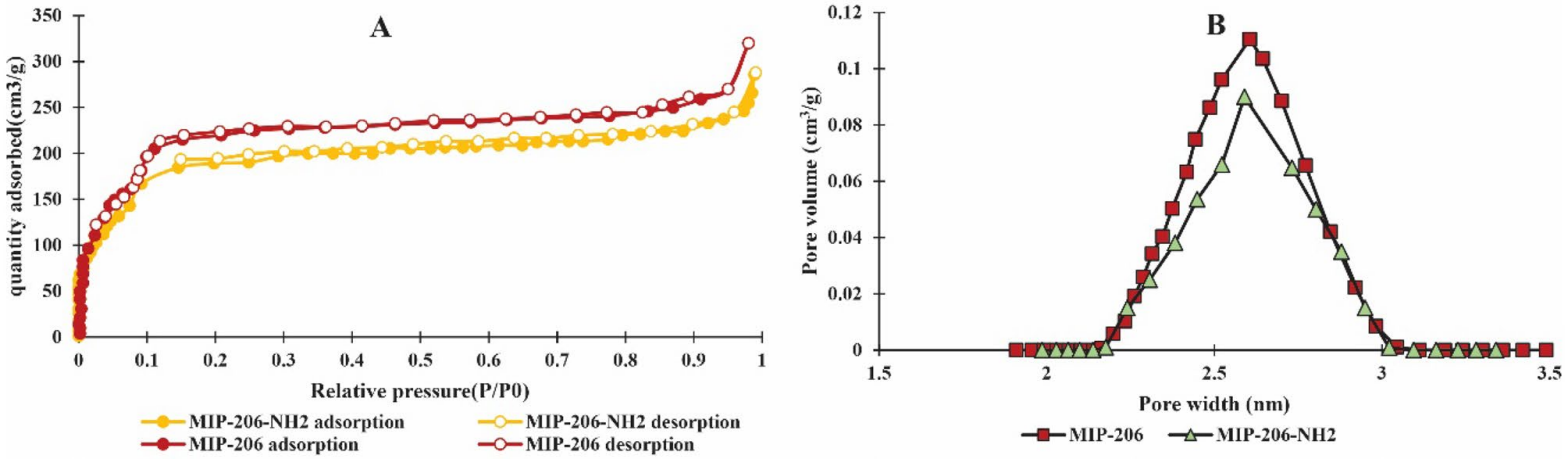
N_2_ adsorption–desorption isotherms of MIP-206 and MIP-206-NH_2_ (**a**), pore size distri-bution of MIP-206 and MIP-206-NH_2_ (**b**).

Figure 6 illustrates the N_2_ adsorption–desorption patterns and pore diameter distributions for both MIP-206 and MIP-206-NH_2_. As seen in Fig. 6a, both patterns exhibit characteristics typical of Type IV isotherms ^40^. This type of isotherm is commonly associated with mesoporous materials characterized by a relatively uniform pore size distribution^40,41^. During the adsorption process, nitrogen molecules gradually fill the mesopores, leading to a steep increase in the adsorbed quantity with an elevation in relative pressure. The hysteresis loop observed during desorption re-sults from the release of nitrogen molecules from the mesopores, leading to a gradual decrease in the adsorbed quantity^40^. While MIP-206 demonstrates a surface area of 1345.21 m^2^/g, MIP-206-NH_2_ exhibits a relatively smaller surface area of 1169.86 m^2^/g. This reduction in surface area is related to the incorporation of amine moieties into the pore network. Additionally, the pore diameter distribution data indicates a slight decrease in the pore space of MIP-206-NH_2_ due to the incorporation of amines into the pore network.

### XPS

**Figure 7:**
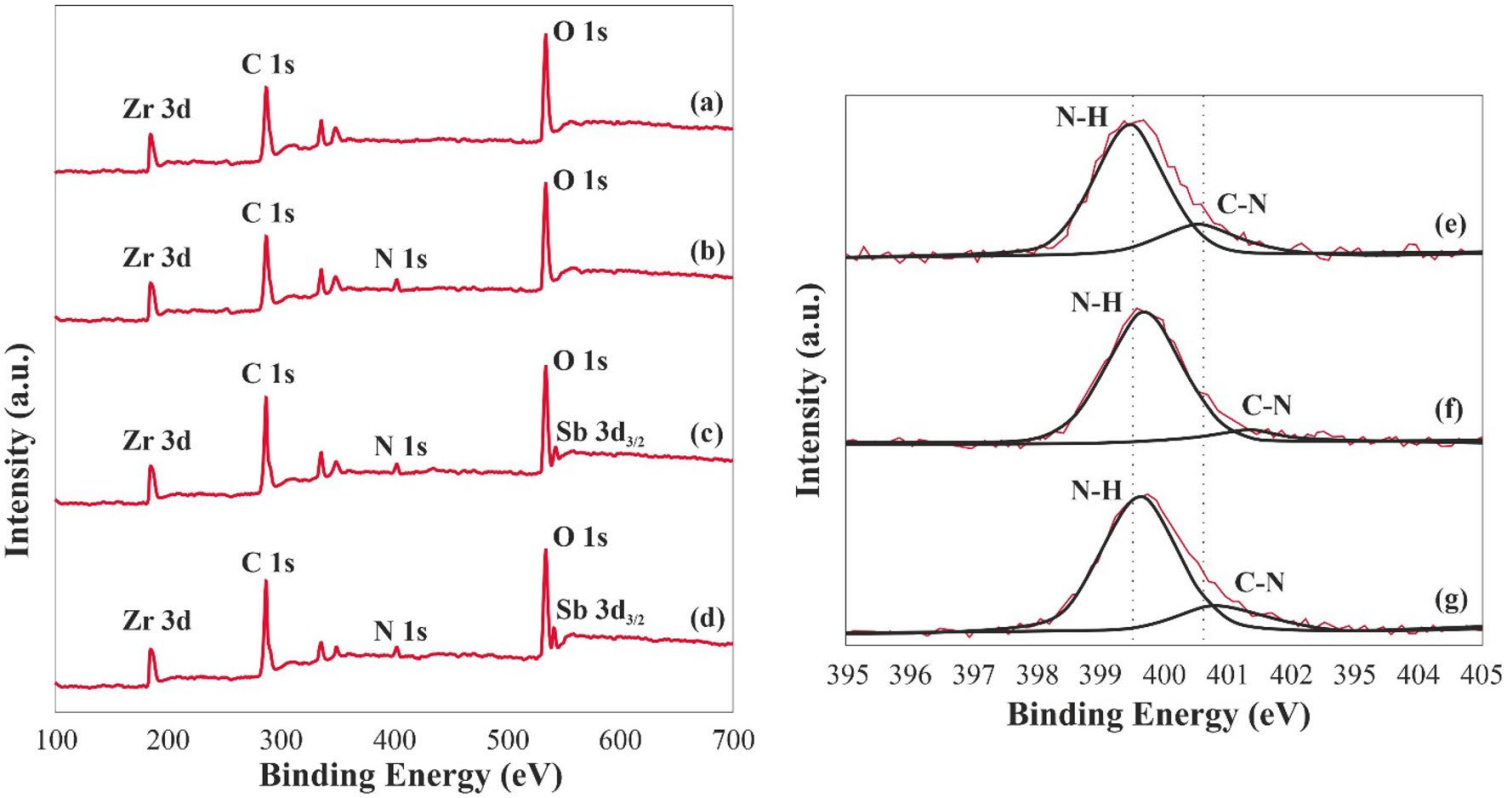
Full spectrum XPS result of (**a**) MIP-206 before antimony separation process, (**b**) MIP-206-NH_2_ before antimony separation process, (**c**) MIP-206-NH_2_ after Sb (III) separation process, (**d**) MIP-206-NH_2_ after Sb (V) separation process, (**e**) high-resolution spectrum of N 1s for MIP-206-NH_2_ before antimony separation process, (**f**) high-resolution spectrum of N 1s for MIP-206-NH_2_ after Sb (III) separation process, and (**g**) high-resolution spectrum of N 1s for MIP-206-NH_2_ after Sb (V) separation process.

To evaluate the integration of amine groups and their interactions with antimony ions, XPS results of MIP-206 and MIP-206-NH2 before and after the antimony separation process are depicted in Fig. 7. The full-scan spectrum of MIP-206 and MIP-206-NH2 after the separation process reveals peaks corresponding to Sb, C, O, and Zr, indicating the presence of antimony on both adsorbents. The peaks attributed to C 1*s* at 285.01 eV remain unchanged after the antimony separation process. However, the signals attributed to N 1*s* at 400.91 and 399.62 eV exhibit slight shifts after the separation process, suggesting the involvement of amine groups in the antimony ion separation process.

### Sb separation performance of MIPs from synthetic wastewater media

**Figure 8:**
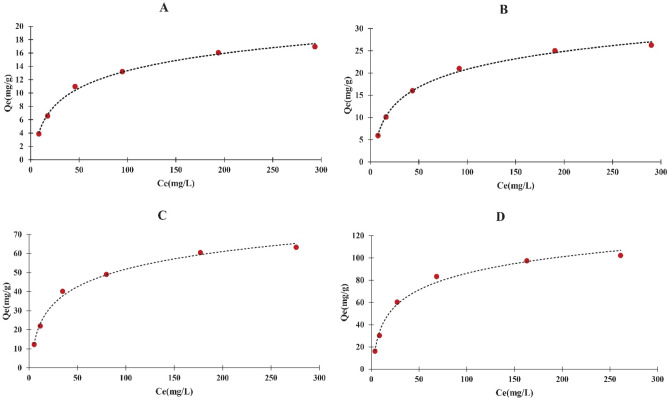
Impact of Sb content on separation performance of MIPs and their Langmuir isotherm fitting; (**A**) Sb (III) concentration vs. MIP-206 separation capacity, (**B**) Sb (V) concentration vs. MIP-206 separation capacity, (**C**) Sb (III) concentration versus MIP-206-NH_2_ separation capacity, (**D**) Sb (V) concentration vs. MIP-206-NH_2_ separation capacity.

Figure 8 illustrates the Sb separation performance of MIP-206 and MIP-206-NH_2_ in synthetic wastewater at varying concentrations of Sb. The graph depicts an increase in the separation capacity of MIPs with rising Sb concentrations, reaching a saturation point for MIPs. Notably, MIP-206-NH_2_ exhibited significantly higher separation capacity (102.18 ± 0.01 mg/g for Sb (V) and 63.23 ± 0.01 mg/g for Sb (III)) compared to MIP-206 (26.26 ± 0.01 mg/g for Sb (V) and 16.95 ± 0.01 mg/g for Sb (III)), attributed to the presence of amine moieties serving as active sites for Sb binding. Freundlich and Langmuir equations were utilized to examine the separation equilibrium data, which are provided in Eqs. (2) and (3).

2 $$ Q_{e} = \frac{{C_{e} K_{l} Q_{m } }}{{1 + C_{e} K_{l} }} $$

3 $$ Q_{e} = K_{f} C_{e}^{1/n} $$

Here, Q_e_(mg/g) denotes the separation capacity of MIPs, Ce(mg/L) is the Sb content at equilibrium, Q_m_(mg/g) is the theoretical separation capacity of MIPs, K_f_(mg/g), n, and K_l_ (L/mg) are the constants of Freundlich and Langmuir equations, respectively. The constants obtained from these equations for Sb (V) and Sb (III) are shown in Table 1.

**Table 1:** Parameters calculated from Langmuir and Freundlich equations.

		${q}_{m}$(mg/g) actual	Langmuir			Freundlich		
			${q}_{m}$(mg/g)	${K}_{l}$(L/mg)	${R}^{2}$	$n$	${K}_{f}$(mg/g)	${R}^{2}$
Sb (III)	MIP-206-NH_2_	63.23 ± 0.01	68.97	0.04	0.99	2.44	7.53	0.91
	MIP-206	16.95 ± 0.01	18.83	0.031	0.99	2.44	1.91	0.93
Sb (V)	MIP-206-NH_2_	102.18 ± 0.01	111.11	0.044	0.99	2.31	11.31	0.89
	MIP-206	26.26 ± 0.01	28.99	0.033	0.99	2.47	3.092	0.92

As per Table 1, the correlation parameter of the Langmuir equation surpasses that of the Freundlich equation, suggesting a better match between equilibrium data and Langmuir equation. Additionally, the theoretically calculated separation capacities for MIPs align well with the experimental values. Consequently, these results suggest that the binding of Sb to MIPs occurs as a monolayer onto energetically equivalent active sites, as described by the Langmuir equation^42^. Table 2 compares some recently investigated materials for antimony separation with the MOFs studied in the current work. The amine-modified MIP-206 (MIP-206-NH2) exhibited superior performance for the separation of both Sb(III) and Sb(V). For instance, while PVA-Fe exhibited a capacity of only 6.99 mg/g and 1.65 mg/g for Sb(III) and Sb(V), respectively, MIP-206-NH2 demonstrated significantly higher capacities of 63.23 mg/g and 102.18 mg/g for Sb(III) and Sb(V), respectively. Overall, the performance of the MOFs developed in this study was much better than the other materials studied, with only ZCN surpassing MIPs in the case of Sb(III).

**Table 2:** Comparision of recently developed adsorbents for antimony removal with materials developed in the current study.

Adsorbent	Adsorption capacity (mg/g)		References
	Sb (III)	Sb (V)	
PVA-Fe	6.99	1.65	^24^
ZCN	70.83	57.17	^25^
Mn-MEP	76.50	–	^43^
NaY@Ce	24.65	7.28	^44^
Fe3O_4_-decorated iron oxy-hydroxides	0.27	0.45	^45^
MIP-206	16.95	26.26	Current study
MIP-206-NH_2_	63.23	102.18	Current study

### Process kinetics

To further understand the process mechanism, process kinetics data was analyzed using pseudo-first and pseudo-second order equations which are presented in Eqs. (4) and (5), respectively.

4 $$ Ln\left( {Q_{m} - Q_{t} } \right) = LnQ_{m} - K_{1} \times t $$

5 $$ {t \mathord{\left/ {\vphantom {t {Q_{t} }}} \right. \kern-0pt} {Q_{t} }} = {1 \mathord{\left/ {\vphantom {1 {K_{2} \times Q_{m}^{2} }}} \right. \kern-0pt} {K_{2} \times Q_{m}^{2} }} + {t \mathord{\left/ {\vphantom {t {Q_{m} }}} \right. \kern-0pt} {Q_{m} }} $$

**Figure 9:**
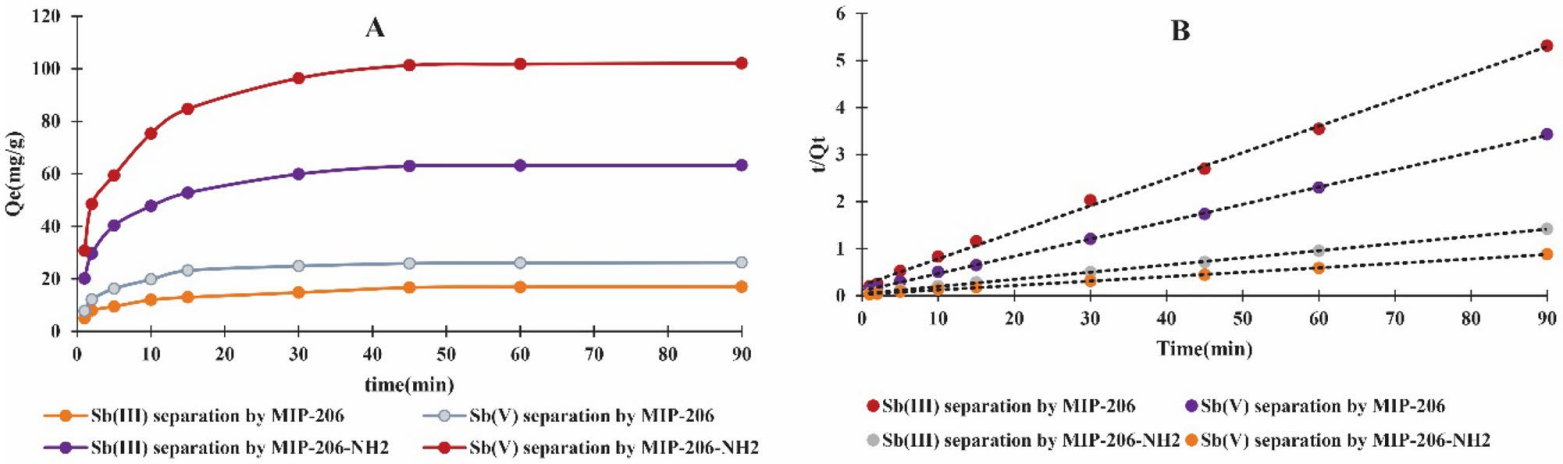
(**A**) Effect of equilibrium time on the separation performance of MIPs, (**B**) pseudo-second order fittings to kinetics data.

Here, ${Q}_{m}$(mg/g) is the theoretical separation capacity, ${Q}_{t}$ (mg/g) is the experimental separation capacity after time $t$, ${K}_{1}$(1/min) and ${K}_{2}$(g/mg.min) are the parameters of kinetic models. Figure 9 and Table 3 present the kinetic data as well as the parameters calculated from the kinetic equations.

**Table 3:** Kinetic parameters of pseudo-first order and pseudo-second order equations.

Pollutants	Materials	Pseudo-first-order			Pseudo-second-order		
		${q}_{m}$(mg/g)	*K*_*1*_ (1/min)	*R*^*2*^	${q}_{m}$(mg/g)	*K*_*2*_ (g/mg.min)	*R*^*2*^
Sb (III)	MIP-206-NH_2_	39.71	0.095	0.961	65.79	0.005	0.999
	MIP-206	12.06	0.086	0.955	17.7	0.015	0.998
Sb (V)	MIP-206-NH_2_	58.77	0.079	0.965	106.38	0.003	0.999
	MIP-206	15.24	0.081	0.97	27.17	0.012	0.999

Figure 9 depicts that both MIPs have similar equilibrium time and their separation capacity reaches a plateau at a very short time of approximately 43 min. The well-distributed and highly accessible pore network within the Zr-based MOFs facilitates rapid diffusion of Sb(V) and Sb(III) ions into the pores. The uniform pore size distribution in the range of 2–3 nm ensures that the adsorbate molecules can quickly navigate through the material without significant resistance. This network provides numerous adsorption sites that are readily available for binding antimony species, contributing to the rapid uptake. MOFs have a large surface area, which increases the number of active sites available for adsorption. This high surface area, combined with the optimal pore size, maximizes the contact between the antimony ions and the adsorbent, leading to faster sorption kinetics. Furthermore, in comparison with the pseudo-first order equation, the correlation parameter of the pseudo-second order equation was greater, suggesting that this equation better predicts the kinetics of Sb separation from waste media. This better fit to the pseudo-second order model suggests that this process is not solely dependent on physical forces (such as diffusion) but involves a significant contribution from chemical in-teractions between Sb and MIPs^46^. The results reveal that MIP-206-NH_2_ demonstrated significantly higher capacity compared to other materials documented in the literature^47–49^. Moreover, MIP-206-NH_2_ reached its maximum capacity in a notably shorter time compared to previously reported materials, underscoring the superior performance of MIP-206-NH_2_ in Sb separation.

### pH effect

**Figure 10:**
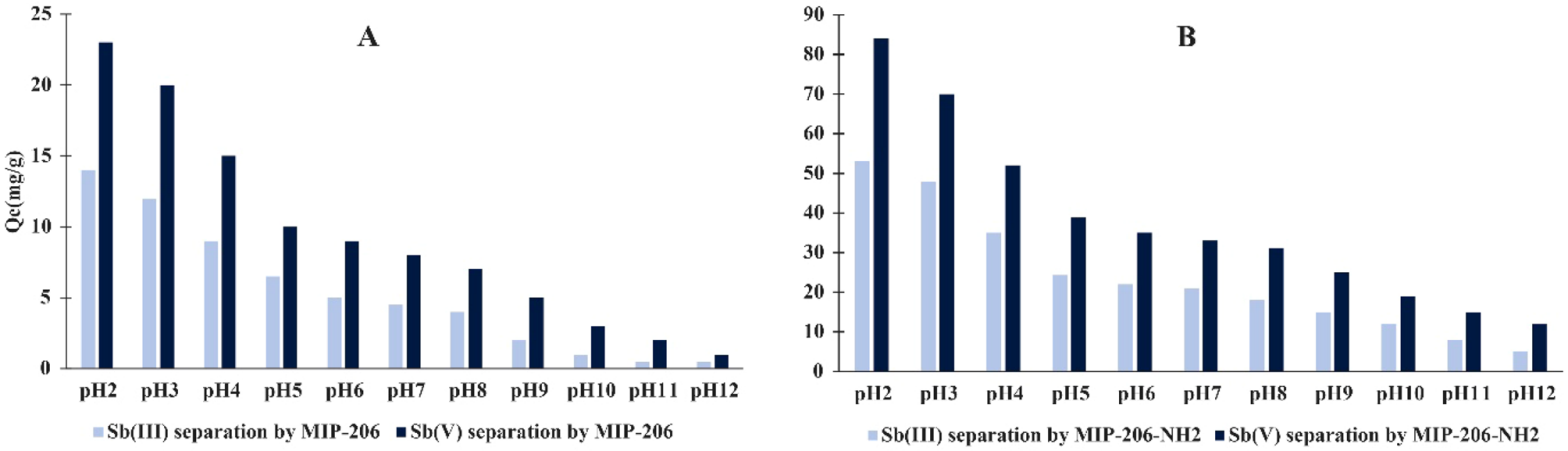
pH performance of (**A**) MIP-206, and (**B**) MIP-206-NH_2_.

Figure 10 illustrates the influence of media pH on the Sb separation process. The optimal separation performance was observed at pH 2, with a gradual decrease in the Sb (V) and Sb (III) separaion as the pH increased. The point of zero charge (pHpzc) was measured to be 7.3 for MIP-206 and 8.6 for MIP-206-NH_2_. Below pHpzc, the protonation reaction rendered the surface charge of MIPs positive, whereas above this value, the surface charge became negative. In the pH range of 2–10.4, the dominant speciation for Sb (V) and Sb (III) are Sb(OH)^-6^ and Sb(OH)_3_, respectively^50^. Consequently, at lower pH values, the attractive forces between Sb compounds and the MIP surface result in enhanced separation performance. Conversely, at alkaline pH levels, repulsive interactions lead to a significant decrease in separation performance. It is noteworthy that waste media containing Sb typically has an acidic pH, making it a suitable environment for MIPs to effectively separate Sb (V) and Sb (III)^51,52^.

### Recovery of Sb from MIPs

**Figure 11:**
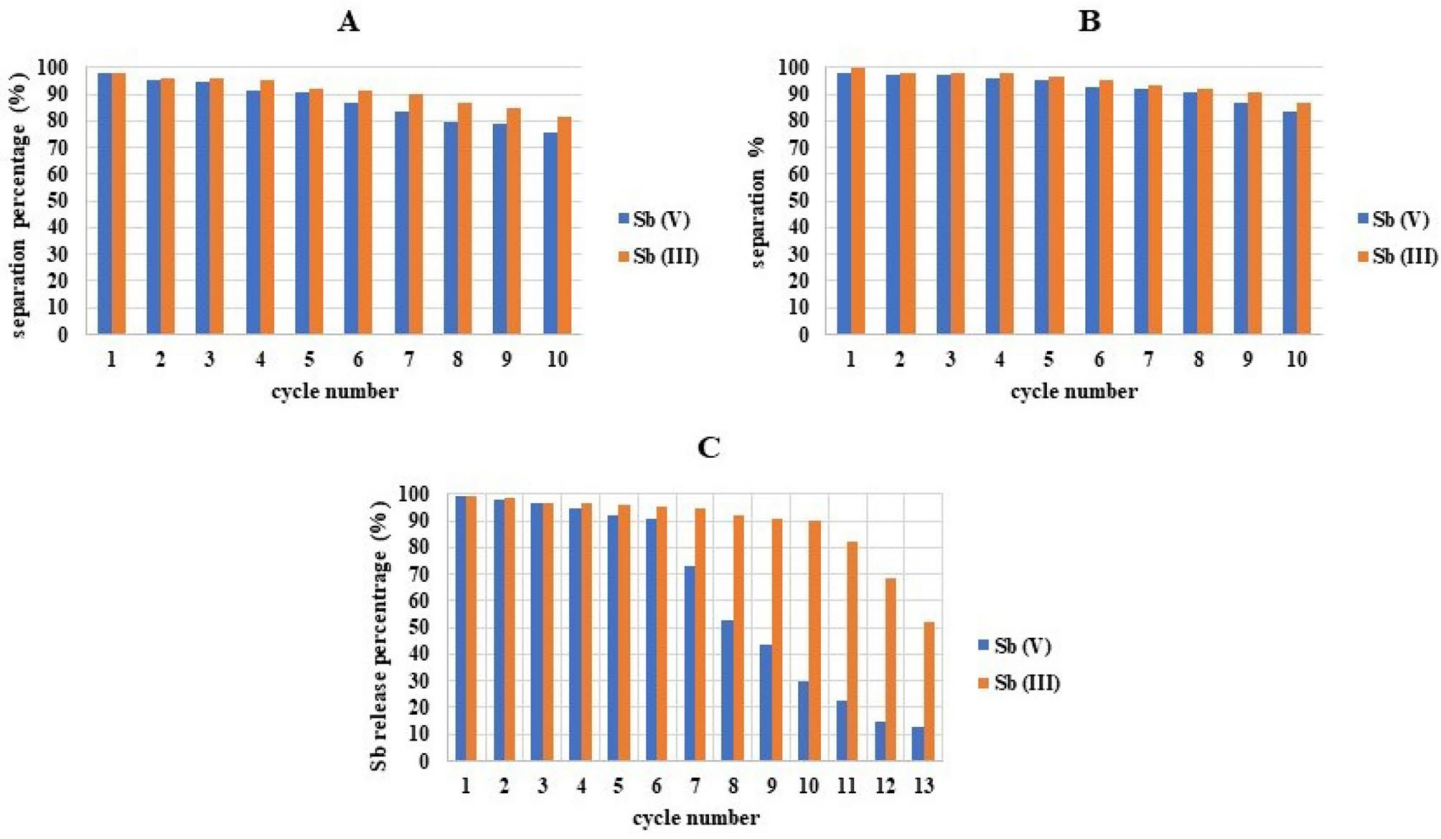
Reusability performance of (**A**) MIP-206-NH_2_, and (**B**) MIP-206 over 10 cycles; (**C**) performance of regeneration solution.

In an industrial context, the ability to reuse MIPs and obtain an Sb rich solution is of significant importance. Figure 11a,b illustrate the recovery performance of MIPs over 10 cycles. Remarkably, MIP-206-NH_2_ demonstrated the capability to retain nearly 90% of its maximum capacity for up to 9 cycles, whereas MIP-206 maintained its performance for approximately 7 cycles. This observation suggests that in a potential continuous system, MIPs can be employed for numerous cycles without experiencing significant exhaustion. The observed difference in cycling stability between MIP-206-NH_2_ and MIP-206 may be attributed to the enhanced affinity of MIP-206-NH_2_ towards Sb(V) and Sb(III) species. The introduction of amino functional groups (-NH_2_) during the modification process could potentially increase the availability of active sites or improve the accessibility of these sites to the target ions, leading to more efficient and stable separation performance. The gradual decrease in sorption capacity observed in the cyclic tests could be attributed to several factors, including saturation of active sites, pore blockage, or structural changes in the adsorbent material over repeated cycles. However, given the stability of the MIPs and the absence of structural changes after the separation process, the primary factors influencing the separation process are likely to be either pore blockage or active site saturation.

Figure 11c illustrates the performance of the regeneration solution in recovering Sb from MIPs. According to the figure, the regeneration solution exhibits a capacity of approximately 0.63 mol/L for Sb (V) and 0.71 mol/L for Sb (III). The recovery of Sb from the surface of MIPs involves the replacement of Sb on the surface of MIPs by hydroxide ions (OH^−^) from the solution^16^. For instance, in the case of Sb (V) separation via MIP-206-NH_2_, the regeneration solution can recover up to 90% of the Sb bound to the surface of MIP-206-NH_2_ for 6 cycles, after which its performance experiences a significant decline. This decline is attributed to the competition between the already existing Sb (V) content in the solution and OH^-^ species to bind to the surface of MIP-206-NH_2_. Therefore, the amount of released Sb before the noticeable drop in performance was utilized to calculate the capacity of the regeneration solution.

### Sb separation from real waste media and selectivity analysis

Table 4 presents the metal composition of the actual mining wastewater employed in this study. MIP-206 and MIP-206-NH_2_ exhibited outstanding efficacy in separating Sb from the waste media. In the pH range of 2–10.4, the dominant speciation for Sb(V) and Sb(III) are Sb(OH)^−6^ and Sb(OH)^−3^, respectively. Consequently, Sb species exist primarily in an anionic form under acidic pH conditions of the wastewater sample. The positive surface charge of the cationic MOFs provides an ideal environment for the adsorption of Sb species. On the other hand, other metal species present in the sample exist in either a cationic form or neutral form of Cd^2+^, Pb^2+^, and Sn(OH)_4_, leading to repulsive electrostatic interactions with the positive surface of the MOFs, thus hindering their adsorption. This selectivity arises from the electrostatic repulsion between the cationic species and the positively charged surface of the MOFs, thereby enabling the preferential removal of Sb species from the solution. However, it was observed that some of the As also adhered to the surface of MIPs. This phenomenon can be attributed to the prevailing speciation of As, primarily in the form of As (V) as H_2_AsO^−^_4_, within the acidic pH range^53^. This form of arsenic is attracted to the positively charged surface of MIP-206-NH_2_.

**Table 4:** Composition of a real mining wastewater before and after Sb separation process at original wastewater pH of 3.40.

	Sn (mg/L)	Pb (mg/L)	Cd (mg/L)	Sb (mg/L)	As (mg/L)
Before	2.61 ± 0.01	2.08 ± 0.01	1.24 ± 0.01	2.48 ± 0.01	2.91 ± 0.01
After	2.54 ± 0.01	1.98 ± 0.01	1.21 ± 0.01	0.08 ± 0.01	2.24 ± 0.01

## Conclusions

In summary, this research highlights the potential of MIP-206 and MIP-206-NH_2_ as promising options for the recovery of both Sb (V) and Sb (III) from mining waste media. The maximum separation capacities were observed as 26.6 mg/g for Sb (V) and 16.95 mg/g for Sb (III) for MIP-206, and 102.18 mg/g and 63.23 mg/g for Sb (V) and Sb (III), respectively, for MIP-206-NH_2_. The Langmuir equation and the pseudo-second-order kinetic model both provided an effective representation of the separation process. Both MIPs demonstrated remarkable reusability, with MIP-206 maintaining continuous performance for 7 cycles and MIP-206-NH_2_ for 9 cycles. Moreover, they exhibited successful Sb recovery from real mining waste media. SEM and XRD analyses confirmed that amine modification did not compromise the structural integrity of MIP-206-NH_2_, while the surface area slightly decreased from 1345.21 to 1169.86 m^2^/g. Overall, MIP-206 and MIP-206-NH_2_ emerge as promising candidates for efficient Sb recovery, showcasing their potential for practical application in the management of mining waste media.

## Data Availability

The raw data supporting the conclusions of this article will be made available by the corresponding author (Jie Liao; jieliaoresearchcenter@gmail.com) on request.
